# Bioluminescence of (*R*)-Cypridina Luciferin with *Cypridina* Luciferase

**DOI:** 10.3390/ijms25052699

**Published:** 2024-02-26

**Authors:** Shusei Kanie, Chun Wu, Kiyohito Kihira, Rie Yasuno, Yasuo Mitani, Yoshihiro Ohmiya

**Affiliations:** 1 Bioproduction Research Institute, National Institute of Advanced Industrial Science and Technology (AIST), Hokkaido Center, 2-17-2-1 Tsukisamu-Higashi, Toyohira-ku, Sapporo 062-8517, Japan; 2 Biomedical Research Institute, AIST, Kansai Center, 1-8-31 Midorigaoka, Ikeda 563-8577, Japan; 3 Japan Aerospace Exploration Agency (JAXA), Tsukuba Space Center, 2-1-1 Sengen, Tsukuba 305-8505, Japan; 4 Cellular and Molecular Biotechnology Research Institute, AIST, Tsukuba Center, 1-1-1 Higashi, Tsukuba 305-8566, Japan; 5 Department of Biomedical Engineering, Osaka Institute of Technology (OIT), 5-16-1 Ohmiya, Asahi-ku, Osaka 535-8585, Japan

**Keywords:** Cypridina luciferin, bioluminescence, marine natural product, *Cypridina* luciferase, chiral separation

## Abstract

Cypridina luciferin (CypL) is a marine natural product that functions as the luminous substrate for the enzyme *Cypridina* luciferase (CypLase). CypL has two enantiomers, (*R*)- and (*S*)-CypL, due to its one chiral center at the *sec*-butyl moiety. Previous studies reported that (*S*)-CypL or racemic CypL with CypLase produced light, but the luminescence of (*R*)-CypL with CypLase has not been investigated. Here, we examined the luminescence of (*R*)-CypL, which had undergone chiral separation from the enantiomeric mixture, with a recombinant CypLase. Our luminescence measurements demonstrated that (*R*)-CypL with CypLase produced light, indicating that (*R*)-CypL must be considered as the luminous substrate for CypLase, as in the case of (*S*)-CypL, rather than a competitive inhibitor for CypLase. Additionally, we found that the maximum luminescence intensity from the reaction of (*R*)-CypL with CypLase was approximately 10 fold lower than that of (*S*)-CypL with CypLase, but our kinetic analysis of CypLase showed that the K_m_ value of CypLase for (*R*)-CypL was approximately 3 fold lower than that for (*S*)-CypL. Furthermore, the chiral high-performance liquid chromatography (HPLC) analysis of the reaction mixture of racemic CypL with CypLase showed that (*R*)-CypL was consumed more slowly than (*S*)-CypL. These results indicate that the turnover rate of CypLase for (*R*)-CypL was lower than that for (*S*)-CypL, which caused the less efficient luminescence of (*R*)-CypL with CypLase.

## 1. Introduction

**Figure 1:**
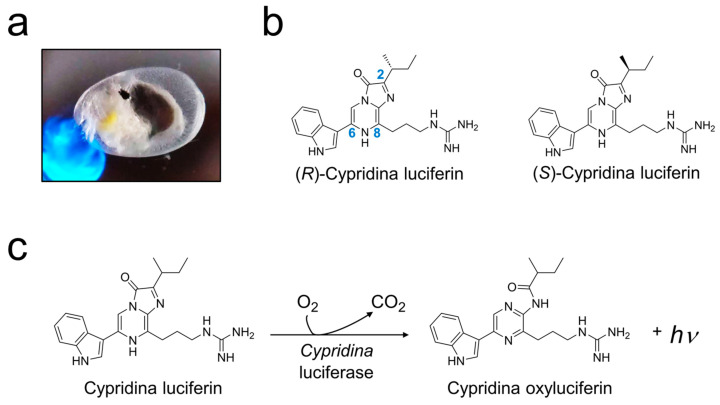
Luminescence reaction of Cypridina luciferin (CypL) with *Cypridina* luciferase (CypLase). (**a**) A photo of the bioluminescence of a *Cypridina* specimen including CypL. The photo was taken on a smartphone (AQUOS SH-01M; Sharp, Osaka, Japan) equipped with a mobile microscope (L-eye; Science Communication Research Institute/SCRI, Kanagawa, Japan). (**b**) Chemical structures of (*R*)- and (*S*)-CypL. (**c**) Luminescence reaction scheme of CypL with CypLase.

Cypridina luciferin (CypL, IUPAC name: 2-[3-[2-[(2*S*)-butan-2-yl]-3-hydroxy-6-(1*H*-indol-3-yl)imidazo [2,1-c]pyrazin-8-yl]propyl]guanidine) is a chiral imidazopyrazinone compound and is responsible for blue light emission from luminous ostracods of the family Cypridinidae, commonly known as sea firefly or marine firefly in English, and umi-hotaru in Japanese (Figure 1a,b) [1,2,3]. In the bioluminescence system, CypL reacts with molecular oxygen to produce light, Cypridina oxyluciferin (CypOxyL), and CO_2_ (Figure 1c) [4]. The luminescence reaction requires the specific enzyme *Cypridina* luciferase (CypLase) to proceed and, thus, many efforts have been made to enzymologically understand the luminescence reaction of CypL with CypLase. CypLase genes have been cloned from two different species of luminous ostracods, *Vargula* (*Cypridina*) *hilgendorfii* and *Cypridina noctiluca* [5,6,7,8,9,10], and recent transcriptome analyses of luminous ostracods that inhabit the Caribbean Sea and the coast of California showed putative CypLase genes [11]. These CypLase genes share sequence similarity, and the reported recombinant CypLases can use CypL as their luminous substrate. Furthermore, different heterologous expression systems for CypLase have been developed [5,8,12,13,14,15], and various mutagenesis studies of CypLase with a focus on subjects such as active amino acid residues, glycan modification, and bioluminescence color have been reported [15,16,17]. However, the detailed catalytic mechanism of CypLase, especially its chirality-recognition ability, remains to be clarified.

To understand the substrate recognition of CypLase, the structure–activity relationship has been investigated with a focus on the side-chain moieties at the C2, C6, and C8 positions in the imidazopyrazinone ring of CypL (Figure 1b). Goto reported that the light yield of CypL with CypLase was dramatically decreased by substitution of the indole ring moiety at the C6 position with a phenyl ring moiety or a change of length of the alkyl guanidine moiety at the C8 position [18]. Additionally, Nakamura et al. reported that CypL analogs that have an oxygen or a sulfur atom instead of the NH group in the indole ring moiety at the C6 position produced light with lower efficiencies in the presence of CypLase [19]. On the other hand, Wu et al. showed that the substitution of the *sec*-butyl moiety at the C2 position with a propyl moiety retained 67% of the original light yield [20]. This result suggests that the recognition of the *sec*-butyl moiety in CypLase for the luminescence reaction is not very strict, despite the existence of one chiral center in the *sec*-butyl moiety of CypL. However, the luminescence of the (*R*)-enantiomer, (*R*)-CypL (IUPAC name: 2-[3-[2-[(2*R*)-butan-2-yl]-3-hydroxy-6-(1H-indol-3-yl)imidazo [2,1-c]pyrazin-8-yl]propyl]guanidine), with CypLase has not yet been compared with that of (*S*)-CypL with CypLase. Furthermore, characterization of the luminescence of (*R*)-CypL with CypLase is more essential for understanding the ability of CypLase to recognize the chirality of CypL for light emission than previous studies with a focus on the luminescence of CypL analogs. Therefore, the ability of CypLase to recognize the chirality of CypL for light emission is still unclear.

Following the intensive efforts of various groups in the first half of the twentieth century to study the bioluminescence of a *Cypridina* specimen [21,22], Shimomura et al. finally succeeded in the isolation and crystallization of CypL from pulverized dry *Vargula* (*Cypridina*) *hilgendorfii* in 1957 [23]. The chemical structure with an imidazopyrazinone skeleton and one chiral center was determined by chemical synthesis [24,25,26,27]. The absolute configuration of the *sec*-butyl moiety of natural CypL was determined to be (*S*) according to analysis using Cypridina hydroluciferin, which was obtained by hydrogenation of natural CypL, and d- and l-amino acid oxidases [24]. In addition, previous studies showed that the light yields of both natural CypL and chemically synthesized (*S*)-CypL with CypLase were approximately 160% of that of racemic CypL with CypLase [19,20,28], suggesting that natural CypL is not a racemic mixture but an (*S*)-enantiomer. Notably, CypL can be biosynthesized from l-arginine, l-tryptophan, and l-isoleucine in living *V. hilgendorfii* specimens [29,30,31,32]. Kato et al. showed that l-isoleucine, which had an absolute configuration identical to that of the *sec*-butyl moiety of (*S*)-CypL, was stereoselectively incorporated into CypL in an in vivo incorporation experiment [31]. However, none of the literature has reported the conditions for chiral high-performance liquid chromatography (HPLC) separation of the enantiomeric mixture of CypL, and there has been no report confirming the absence of (*R*)-CypL in nature. Furthermore, although the function of the enantiomers of chiral luciferins (e.g., firefly luciferin) in some bioluminescence systems has attracted attention, no report reveals whether (*R*)-CypL functions as a luminous substrate or an inhibitor for CypLase.

In this study, to confirm whether the enantiomer of (*S*)-CypL functions as a luminous substrate for CypLase, we examined the luminescence of (*R*)-CypL, which had undergone chiral separation from the enantiomeric mixture, with a recombinant CypLase. As a result, we demonstrated for the first time that (*R*)-CypL functions as the luminous substrate for CypLase, and our kinetic analysis of CypLase suggests a higher affinity of (*R*)-CypL for CypLase than (*S*)-CypL. Additionally, we found that the turnover rate of CypLase for (*R*)-CypL was different from that of CypLase for (*S*)-CypL, which would be expected to cause the luminescence of (*R*)-CypL with CypLase to become less efficient than that of (*S*)-CypL with CypLase.

## 2. Results

### 2.1. Chiral Separation of (R)-CypL from the Enantiomeric Mixture

To prepare (*R*)-CypL, we had two options: the asymmetric synthesis or the chiral separation of the enantiomeric mixture of CypL. Following the first total synthesis of CypL by Kishi et al., other methods have been developed for the chemical synthesis of racemic CypL or (*S*)-CypL [19,20]. However, as far as we know, chiral separation of the enantiomeric mixture of CypL has not yet been reported. Therefore, we tried to prepare (*R*)-CypL by chiral separation using a chiral column.

**Figure 2:**
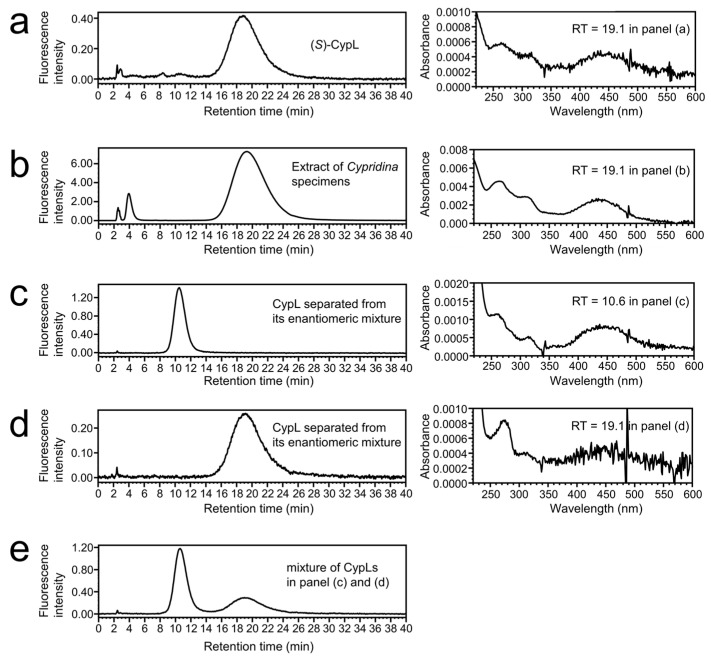
Chiral HPLC analysis of (*R*)- and (*S*)-CypL used in luminescence measurements. HPLC chromatograms with fluorescence detection (excitation wavelength, 430 nm; emission wavelength, 570 nm) and absorption spectra were simultaneously obtained via PDA detection in the chiral HPLC analyses. (**a**) Chemically synthesized (*S*)-CypL (185 pmol, 75.0 ng) at a retention time (RT) of 19.1 min, (**b**) methanolic extract from ten dried luminous ostracods at a RT of 19.1 min, (**c**) chiral-separated CypL (93.5 pmol, 37.9 ng), which was initially eluted under our chiral HPLC conditions (see Figure A1 and Section 4 “Chiral HPLC Separation of Enantiomers of CypL”) at an RT of 10.6 min, (**d**) chiral-separated CypL (83 pmol, 33.7 ng) which was next eluted in our chiral HPLC separation (see Figure A1 and Section 4 “Chiral HPLC Separation of Enantiomers of CypL”) at an RT of 19.1 min, (**e**) a 1:1 mixture of chiral-separated CypLs from panel c (93.5 pmol, 37.9 ng) and panel d (83 pmol, 33.7 ng). A chiral column CHIRALCEL OZ-RH (ϕ4.6 × 150 mm, 5 μm; Daicel Chemical Industry, Osaka, Japan) was used in this chiral HPLC analysis.

**Figure A1:**
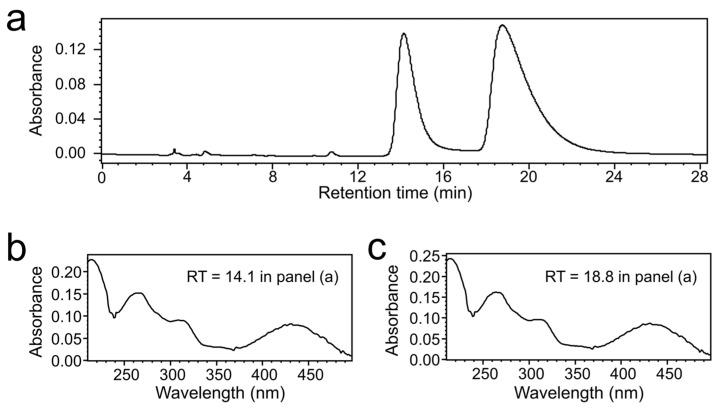
Chiral HPLC separation of the enantiomeric mixture of CypL. (**a**) HPLC chromatogram at 430 nm. Absorption spectra obtained via PDA detection at the retention times of (**b**) 14.1 and (**c**) 18.8 shown in panel (**a**) (see Section 4 “Chiral HPLC Separation of Enantiomers of CypL”). The amount of CypL (NanoLight Technology, Prolume) used in this experiment was 10 μg. A CHIRALPAK IM chiral column (ϕ4.6 × 250 mm, 5 μm; Daicel Chemical Industry) was used in this chiral HPLC analysis.

Based on chiral column screening for racemic CypL, we subjected an enantiomeric mixture of CypL (Supplementary Figure S1 and Table S1) to chiral HPLC separation using chiral columns under solvent conditions that employed a mixture of acetonitrile and 100 mM aqueous solution of potassium hexafluorophosphate (see Section 4 “Chiral HPLC Separation of Enantiomers of CypL”). As a result, we observed two clearly separated peaks in the obtained HPLC chromatogram at 430 nm (Figure A1a). The absorption spectra of the two peaks were identical to each other (Figure A1b,c). To identify which peak was (*R*)-CypL, we next analyzed chemically synthesized (*S*)-CypL and CypL extracted from natural luminous *Cypridina* specimens under the same chiral HPLC conditions. The elution times of the chemically synthesized (*S*)-CypL and the extracted natural CypL corresponded to the latter peak found in the chiral HPLC analysis of the enantiomeric mixture of CypL (Figure 2). The absorption spectra of the two peaks separated from the enantiomeric mixture of CypL were also identical to those of the synthesized (*S*)-CypL and the extracted natural CypL. This result showed that the former peak under our chiral HPLC conditions was (*R*)-CypL and the latter was (*S*)-CypL.

### 2.2. Luminescence Spectrum of (R)-CypL with CypLase

**Figure 3:**
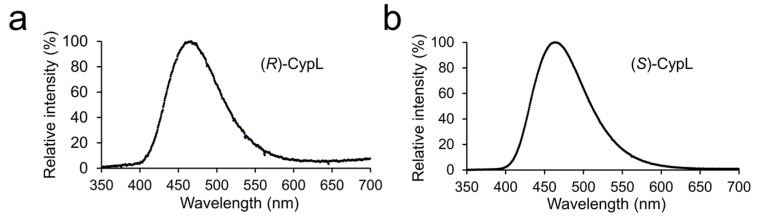
Luminescence emission spectra of (*R*)- and (*S*)-CypL with a recombinant CypLase in 100 mM Tris–HCl (tris(hydroxymethyl)aminomethane–hydrochloric acid, pH 7.5) containing 300 mM l-ascorbic acid sodium salt. (**a**) (*R*)-CypL (40.5 ng) and (**b**) (*S*)-CypL (40.5 ng) with a recombinant CypLase (5 ng).

To test whether (*R*)-CypL functions as a luminous substrate for CypLase, we measured the luminescence spectrum from the reaction of the chiral-separated (*R*)-CypL with a recombinant CypLase (Supplementary Figure S2). The luminescence spectrum was successfully obtained, and the maximum emission wavelength (466 nm) was almost identical to that in the luminescence of (*S*)-CypL with a recombinant CypLase (464 nm) (Figure 3). This result indicates that (*R*)-CypL functioned as a luminous substrate for CypLase, as in the case of (*S*)-CypL.

### 2.3. Luminescence Intensity of (R)-CypL with CypLase

**Figure 4:**
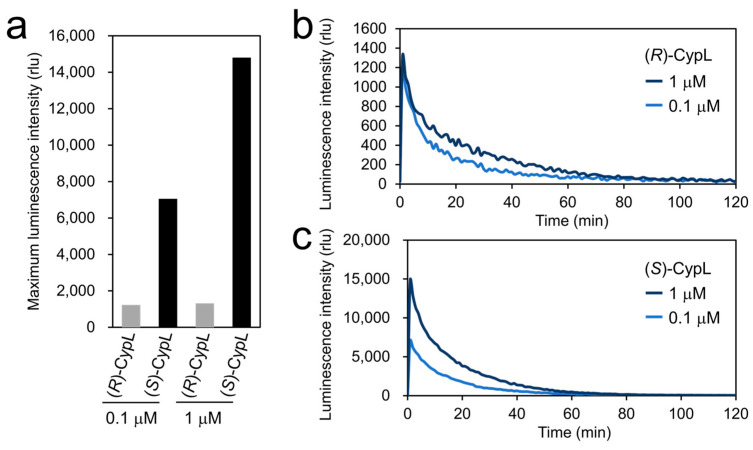
Luminescence of (*R*)- and (*S*)-CypL with a recombinant CypLase. (**a**) Maximum luminescence intensity of CypLs with a recombinant CypLase over 120 min. Luminescence kinetics of (**b**) (*R*)-CypL and (**c**) (*S*)-CypL over 120 min. The amount of CypL used in this measurement was 4.05 ng or 40.5 ng, respectively. The amount of CypLase used in this experiment was 0.5 ng. Relative light unit is abbreviated as rlu.

Next, to characterize the luminescence of (*R*)-CypL with CypLase, we compared the luminescence intensity of (*R*)-CypL with that of (*S*)-CypL via luminescence measurements using a recombinant CypLase. In our luminescence measurements over 120 min using black 96-well plates, the values of the maximum luminescence intensity of (*R*)-CypL with a recombinant CypLase were 1232 (at a final substrate concentration of 0.1 μM) and 1318 (at a final substrate concentration of 1 μM), whereas those of (*S*)-CypL with a recombinant CypLase were 7051 (at a final substrate concentration of 0.1 μM) and 14,804 (at a final substrate concentration of 1 μM) (Figure 4). The time-course change in the luminescence intensity of (*R*)-CypL with a recombinant CypLase was different from that of (*S*)-CypL with a recombinant CypLase (Table A1). Additionally, when we calculated the K_m_ values of CypLase for (*R*)- and (*S*)-CypL based on the Michaelis–Menten equation, the calculation showed that the K_m_ value of CypLase for (*R*)-CypL (0.23 μM) was approximately three fold lower than that for (*S*)-CypL (0.75 μM) (Table A2 and Supplementary Figure S3).

**Figure A2:**
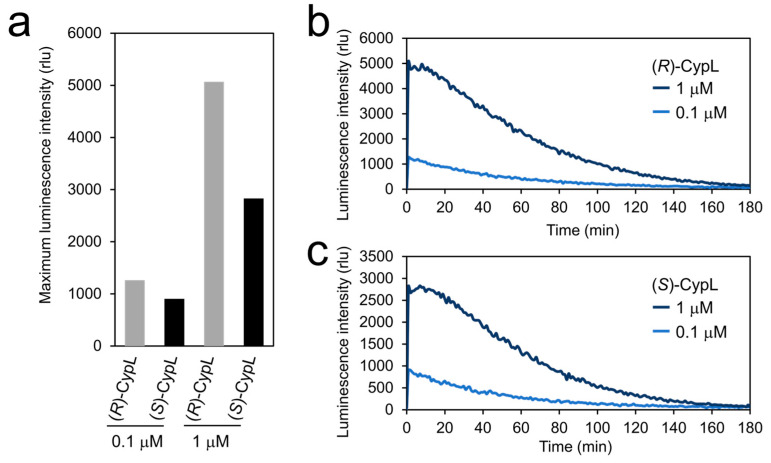
Luminescence of CypL with human AGP. (**a**) Maximum luminescence intensity of CypLs with human AGP over 180 min. Luminescence kinetics of (**b**) (*R*)-CypL and (**c**) (*S*)-CypL with human AGP over 180 min. The amount of CypL used in this measurement was 4.05 ng or 40.5 ng, and the amount of human AGP used in this experiment was 2.65 μg.

Previously, we discovered that human alpha 1-acid glycoprotein (AGP), which is a human plasma glycoprotein, caused the luminescence of racemic CypL [33]. Therefore, we further examined the luminescence intensity of (*R*)- and (*S*)-CypL with human AGP under Tris–HCl (pH 9.0) buffer conditions, which are optimal conditions for the induction of luminescence of CypL by human AGP [33]. Luminescence measurements taken over 180 min using white 96-well plates (see Section 4 “Measurement of Luminescence Intensity of CypLs with Human AGP”) showed that these luminescence kinetics differed from those of (*R*)- and (*S*)-CypL with CypLase. Furthermore, the values of the maximum luminescence intensity of (*R*)-CypL were 1264 (at a final substrate concentration of 0.1 μM) and 5070 (at a final substrate concentration of 1 μM), whereas those of (*S*)-CypL with human AGP were 905 (at a final substrate concentration of 0.1 μM) and 2831 (at a final substrate concentration of 1 μM) (Figure A2). The time-course change in the luminescence intensity of (*R*)-CypL with human AGP was similar to that of (*S*)-CypL with human AGP (Table A3).

Collectively, these results indicate that both (*R*)- and (*S*)-CypL with CypLase and human AGP produced light, but the values of the maximum luminescence intensity of (*R*)- and (*S*)-CypL varied depending on the protein used for the luminescence reaction.

### 2.4. Turnover Rate of CypLase for (R)- and (S)-CypL

**Figure 5:**
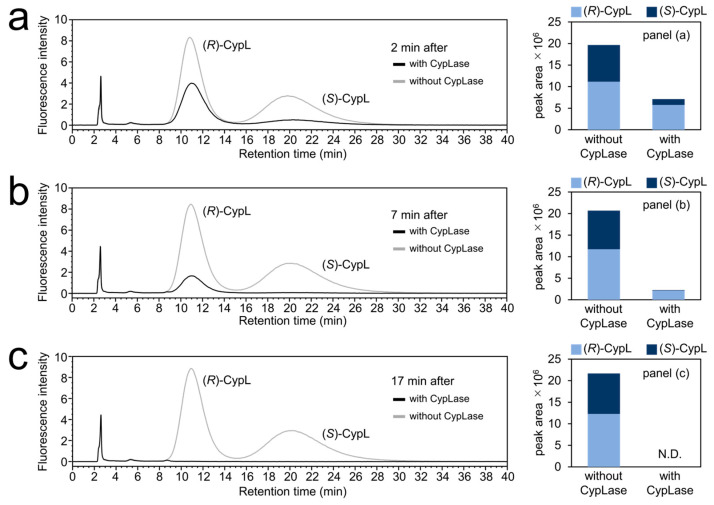
Chiral HPLC analysis of the reaction mixture of racemic CypL with CypLase. Shown are the HPLC chromatograms with fluorescence detection (excitation wavelength, 430 nm; emission wavelength, 570 nm) of the reaction mixture at (**a**) 2 min, (**b**) 7 min, and (**c**) 17 min after the start of the reaction. The initial concentration of CypL was 98.4 μM. The amounts of racemic CypL and CypLase used in this experiment were 23.9 μg and 7.2 μg, respectively. A chiral column CHIRALCEL OZ-RH (ϕ4.6 × 150 mm, 5 μm; Daicel Chemical Industry) was used in this chiral HPLC analysis.

Since the maximum luminescence intensity from the reaction of (*R*)-CypL with a recombinant CypLase was significantly lower than that of (*S*)-CypL with a recombinant CypLase, we next examined whether (*R*)-CypL was consumed more slowly than (*S*)-CypL in the presence of CypLase, via chiral HPLC analysis of the reaction mixture of racemic CypL (initial concentration of 98.4 μM >> the K_m_ values of CypLase for (*R*)-CypL and (*S*)-CypL) with a recombinant CypLase. The chiral HPLC analysis of the reaction mixture at 2 min after starting the reaction in the presence of CypLase showed that the proportions of the peak areas of (*R*)-CypL and (*S*)-CypL to the total CypL were 80.9% and 19.1%, respectively, despite the use of racemic CypL (Figure 5a and Table A4). This result indicated that (*R*)-CypL was consumed more slowly than (*S*)-CypL. At 7 min after starting the reaction in the presence of CypLase, only (*S*)-CypL was almost fully consumed in the presence of CypLase, and the proportions of the peak areas of (*R*)-CypL and (*S*)-CypL to the total CypL were 94.6% and 5.4%, respectively (Figure 5b and Table A4). At 17 min after starting the reaction in the presence of CypLase, neither the peak of (*R*)-CypL nor that of (*S*)-CypL were detected, whereas in the absence of CypLase neither (*R*)- nor (*S*)-CypL in the reaction mixture were consumed (Figure 5c and Table A4). These results indicate that CypLase completely consumed both (*R*)- and (*S*)-CypL with different turnover rates.

## 3. Discussion

In this study, we successfully obtained (*R*)-CypL by chiral HPLC separation of the enantiomeric mixture of CypL instead of asymmetric synthesis (Figure 2 and Figure A1). Although asymmetric synthesis can efficiently provide a large amount of (*R*)- or (*S*)-CypL in a laboratory, the chiral separation of CypL is useful not only for obtaining optically pure CypL but also for analyzing the optical purity of CypL. Until this study, optical rotation measurement was the only way to determine the optical purity of CypL [20]. However, such measurement requires milligrams of CypL, and the optical rotation is affected by the concentration of CypL, the temperature condition and the solvent used in the measurement. In contrast, chiral HPLC analysis with fluorescence detection enables us to analyze the optical purity of a solution of a few micromoles of CypL. In fact, we detected only (*S*)-CypL from a methanolic extract of ten dried luminous ostracods (Figure 2b), suggesting for the first time the absence of (*R*)-CypL in nature. Our method of chiral HPLC analysis of CypL is expected to contribute to studies on the biosynthesis of CypL, since the chiral HPLC analysis of firefly luciferin has been used to study the biosynthesis of firefly luciferin [34,35,36].

In the luminescence measurements of (*R*)-CypL with a recombinant CypLase, we showed that (*R*)-CypL reacted with CypLase to produce light in the same way as the reaction of (*S*)-CypL with CypLase (Figure 3). This result is reasonable based on the fact that the reaction of CypLase with the CypL analog that had a propyl moiety substituted for the original chiral *sec*-butyl moiety at the C2 position of the imidazopyrazinone ring gave 67% of the original light yield, suggesting that the recognition of the *sec*-butyl moiety in CypLase for the luminescence reaction is not especially strict. It is noted that some known luciferins including CypL are chiral compounds, and their chirality is recognized by their corresponding luciferases for the luminescence reaction [37,38,39,40]. For example, in the firefly bioluminescence system, D-firefly luciferin is the substrate for firefly luciferase, whereas the (*R*)-enantiomer (l-firefly luciferin) shows an inhibitory effect on the luminescence reaction of D-luciferin with firefly luciferase [41,42]. Therefore, we can consider CypLase, which uses both (*R*)- and (*S*)-CypL for the luminous substrate, as an exceptional luciferase. However, we found that the maximum luminescence intensity of (*R*)-CypL with a recombinant CypLase was significantly lower than that of (*S*)-CypL with the CypLase (Figure 4).

Given that (1) (*R*)-CypL should have chemical and physical properties identical to those of (*S*)-CypL, and (2) the maximum luminescence intensity of (*R*)-CypL with human AGP was significantly higher than that of (*S*)-CypL with human AGP (Figure A2), we considered that the difference in the maximum luminescence intensity with CypLase resulted from the character of CypLase rather than a property of CypL. In the chiral HPLC analysis of the reaction mixture of racemic CypL with a recombinant CypLase, we found that CypLase consumed (*R*)-CypL more slowly than (*S*)-CypL (Figure 5 and Table A4). This result showed that the turnover rate of CypLase for (*R*)-CypL was lower than that of CypLase for (*S*)-CypL, which would account for the lower maximum luminescence intensity and the difference in the time-course change of the luminescence intensity of (*R*)-CypL with CypLase compared with those of (*S*)-CypL with CypLase (Figure 4 and Table A1). However, we do not exclude the possibility that CypLase modifies the three factors that determine the quantum yield of the bioluminescence—namely, the yield of the high energy intermediate for CypOxyL, the yield of CypOxyL in a singlet excited state, and the yield of the fluorescence quantum yield of CypOxyL, between (*R*)-CypL and (*S*)-CypL—and thereby affects the efficiency of luminescence of (*R*)- and (*S*)-CypL with CypLase [43,44,45,46,47,48,49,50]. In fact, Shimomura reported that the fluorescence of CypOxyL was enhanced in CypLase [51]. In addition, the difference between the inhibitory effects of (*R*)- and (*S*)-CypOxyL on the luminescence reaction of CypL with CypLase might also affect the efficiency of luminescence of (*R*)- and (*S*)-CypL with CypLase.

It is noteworthy that the difference between the maximum luminescence intensities of (*R*)- and (*S*)-CypL with a recombinant CypLase varied depending on the concentration of CypL (0.1 μM or 1 μM) (Figure 4). This result can be explained by the difference in the calculated K_m_ values of CypLase (0.23 μM for (*R*)-CypL and 0.75 μM for (*S*)-CypL) (Table A2). Although the finding that the K_m_ value of CypLase for (*R*)-CypL was lower than that for (*S*)-CypL suggested a higher affinity of (*R*)-CypL for CypLase than (*S*)-CypL, the efficiency of luminescence of (*R*)-CypL with CypLase was lower than that of (*S*)-CypL with CypLase. This result is different from results in previous studies with a focus on CypL analogs that produce light less efficiently, because K_m_ values of such CypL analogs for CypLase were higher than that of (*S*)-CypL with CypLase [19]. The less efficient luminescence of (*R*)-CypL with CypLase is possibly because the luminescence reaction of (*R*)-CypL with CypLase proceeds more slowly than that of (*S*)-CypL with CypLase, or because (*R*)-CypOxyL formed from (*R*)-CypL dissociates more slowly from CypLase to decrease the turnover rate of CypLase for the luminescence reaction in comparison with (*S*)-CypOxyL formed from (*S*)-CypL. This finding suggests that the higher affinity of the substrate for CypLase is not the only important point for engineering of CypLase to produce light more efficiently. In fact, a recent study suggested that the optimization of not only the binding of a substrate to an enzyme but also releasing the product from the enzyme can maximize enzymatic activity [52].

In future work based on our findings, we anticipate that crystallographic characterization of CypLase will help to elucidate why the recognition of the *sec*-butyl moiety in CypLase for the luminescence reaction is not very strict.

## 4. Materials and Methods

### 4.1. Materials

The enantiomeric excess (*S*)-CypL (the enantiomeric mixture of CypL; lot number: L1816) was from NanoLight Technology, Prolume (Pinetop, AZ, USA) (see Supplementary Figure S1 and Table S1). Human AGP (alpha 1-acid glycoprotein form human plasma) and potassium hexafluorophosphate were from Sigma-Aldrich (St. Louis, MO, USA). Tris–HCl buffer, l-ascorbic acid sodium salt, and sodium chloride were from FUJIFILM Wako Pure Chemical Corporation (Osaka, Japan). All materials were used without further purification. Racemic CypL and (*S*)-CypL were prepared according to the method reported previously [19,20]. A recombinant CypLase from *C. noctiluca* was prepared using plant cells according to the method reported previously (see Supplementary Figure S2) [14]. The concentrations of CypL solutions were determined spectrophotometrically using a spectrophotometer (V-660; Jasco, Tokyo, Japan), according to the reported molar absorption coefficient [24]. The concentrations of human AGP and recombinant CypLase solutions were determined by SDS-PAGE analysis or by using the corresponding molar extinction coefficient at 280 nm, as calculated via the peptide property calculator available at https://www.biosyn.com/peptidepropertycalculator/peptidepropertycalculator.aspx (accessed on 6 October 2023).

### 4.2. Chiral HPLC Separation of Enantiomers of CypL

CypL (NanoLight Technology, Prolume) was dissolved in methanol. A 10 uL or 50 uL aliquot was subjected to chiral HPLC separation. Chiral HPLC separation was performed on a Waters ACQUITY UPLC H-Class system (Waters, Milford, MA, USA) equipped with a chiral column CHIRALCEL OZ-RH (ϕ4.6 × 150 mm, 5 μm; Daicel Chemical Industry) and a multiwavelength detector (ACQUITY UPLC PDA eλ detector; Waters), or on a Waters alliance system (2695; Waters) equipped with a CHIRALPAK IM chiral column (ϕ4.6 × 250 mm, 5 μm; Daicel Chemical Industry) and a multiwavelength detector (2996 PDA detector; Waters). The HPLC conditions of the Waters ACQUITY UPLC H-Class system were as follows: mobile phase, 20% (*v*/*v*) acetonitrile in a 100 mM solution of potassium hexafluorophosphate in H2O; flow rate, 1.0 mL min^−1^. The HPLC conditions on the Waters alliance system (see Figure A1) were as follows: mobile phase, 25% (*v*/*v*) acetonitrile in a 100 mM solution of potassium hexafluorophosphate in H_2_O; flow rate, 1.0 mL min^−1^. The eluted fractions containing (*R*)- or (*S*)-CypL were lyophilized for 67 h followed by dissolving in methanol or were subjected to solid-phase extraction using a solid-phase extraction column (Polymer-based HLB, AS ONE, Osaka, Japan) followed by elution with ethanol.

### 4.3. Chiral HPLC Analysis of Chiral-Separated CypLs

The methanolic solutions of chiral-separated (*R*)- and (*S*)-CypL were filtered through a centrifugal filter Ultrafree-MC (0.22 μm; Millipore, Billerica, MA, USA) and diluted 2 fold with methanol or mixed together in a 1:1 ratio. The methanolic extract of dried luminous ostracods was prepared as follows: ten dried luminous ostracods in a commercially available kit for observation of bioluminescence of sea-firefly (Hatenouruma, Tokyo) were homogenized in 200 μL of ice-cold methanol on ice and centrifuged at 14,000× *g* for 3 min at 4 °C followed by filtration through an Ultrafree-MC centrifugal filter (0.22 μm; Millipore). Ten microliter aliquots of these prepared solutions were subjected to chiral HPLC analysis. Chiral HPLC analysis was performed on a Waters ACQUITY UPLC H-Class system (Waters) equipped with a CHIRALCEL OZ-RH chiral column (ϕ4.6 × 150 mm, 5 μm; Daicel Chemical Industry), a multiwavelength detector (ACQUITY UPLC PDA eλ detector; Waters), and a fluorescence detector (ACQUITY FLR detector; Waters). The HPLC conditions were as follows: mobile phase, 30% (*v*/*v*) acetonitrile in a 100 mM solution of potassium hexafluorophosphate in H_2_O; flow rate, 0.8 mL min^−1^; fluorescence detection, excitation/emission, 430/570 nm.

### 4.4. Measurement of Luminescence Emission Spectra

The luminescence emission spectra were measured using a LumiFLspectrocapture high-sensitivity charge-coupled device (CCD) spectrophotometer (AB-1850C; ATTO) with the following settings: measurement mode, single; measurement time, 1 min; slit width, 0.5 mm; camera gain, high; diffraction grating, 150 lines mm^−1^; shutter for measurement, automatic. To 50 μL of a 100 ng mL^−1^ solution of a recombinant CypLase in 100 mM Tris–HCl (pH 7.5) containing 300 mM l-ascorbic acid sodium salt in a 0.2 μL micro-tube (0.2 mL thin-walled tube; Thermo Fisher Scientific, Waltham, MA, USA) was manually added 50 μL of a 2 μM solution of chiral-separated (*R*)- or (*S*)-CypL in 100 mM Tris–HCl (pH 7.5) containing 300 mM l-ascorbic acid sodium salt, followed by immediate measurement of the luminescence emission spectrum at room temperature. The final volume of each solution for measurement of the luminescence emission spectrum was 100 μL.

### 4.5. Measurement of the Luminescence Intensity of CypLs with CypLase

Fifty microliters of a 10 ng mL^−1^ solution of a recombinant CypLase in 100 mM Tris–HCl (pH 7.5) containing 300 mM l-ascorbic acid sodium salt and 100 mM NaCl was automatically added to 50 μL of a 0.2 or 2 μM solution of chiral-separated (*R*)- or (*S*)-CypL in 100 mM Tris–HCl (pH 7.5) containing 300 mM l-ascorbic acid sodium salt and 100 mM NaCl on a black 96-well plate (FIA Plate; Greiner Bio-One, Kremsmunster, Austria) using an injector equipped with a multimode microplate reader (TriStar 5; Berthold Technologies, Bad Wildbad, Germany), followed by immediate measurement of luminescence intensity at room temperature. The luminescence intensity was recorded in relative light units (RLU) in 0.1 s intervals over 120 min.

### 4.6. Measurement of Luminescence Intensity of CypLs with Human AGP

Fifty microliters of a 53 μg mL^−1^ solution of human AGP in 100 mM Tris–HCl (pH 9.0) was automatically added to 50 μL of a 0.2 or 2 μM solution of chiral-separated (*R*)- or (*S*)-CypL in water on a white 96-well plate (Eppendorf microplate 96/F-PP; Eppendorf, Hamburg, Germany) using the injector equipped with the multimode microplate reader (TriStar 5; Berthold Technologies), followed by immediate measurement of luminescence intensity at room temperature. The luminescence intensity was recorded in relative light units (RLU) in 0.1 s intervals over 180 min. As a point of caution, in this measurement, a white 96-well plate was used instead of the black 96-well plate described in Section 4.5. A white 96-well plate reflects light, but a black 96-well plate absorbs light. Therefore, the value of light intensity with a white 96-well plate is higher than that with a black 96-well plate.

### 4.7. Kinetic Analysis of CypLase

The luminescence intensity for the kinetic analysis was recorded on a luminometer (AB2200; ATTO), after adding 40 μL of a recombinant CypLase solution (1 ng mL^−1^ in 150 mM phosphate buffer (pH 7.2) containing 100 mM NaCl to 40 μL of a (*R*)- or (*S*)-CypL ethanol solution (0.0156–2 μM) in 0.1 M phosphate buffer (pH 7.2) with antioxidants (300 mM sodium ascorbate/20 mM Na_2_SO_3_) in a test tube at room temperature. Each measurement was performed in duplicate. The obtained data (see Supplementary Figure S3) were subjected to kinetic analysis using the R program [53] to fit the Michaelis–Menten equation.

### 4.8. Chiral HPLC Analysis of the Reaction Mixtures of Racemic CypL and CypLase

Immediately before this experiment, 594 μL of a 99.4 μM solution of racemic CypL in 100 mM Tris–HCl (pH 7.5) containing 300 mM l-ascorbic acid sodium salt was mixed with 6 μL of water or a 1.2 mg mL^−1^ solution of a recombinant CypLase in 20 mM Tris–HCl (pH 8.0). The mixtures were incubated at room temperature, and 200 μL of each mixture was collected at 2 min, 7 min, and 17 min after starting the reaction, followed by immediate filtration through an Amicon Ultra-0.5 centrifugal filter device with nominal molecular weight limit (NMWL) of 10 KDa (Millipore). Ten aliquots of the resultant filtrates were subjected to chiral HPLC analysis. Chiral HPLC analysis was performed on a Waters ACQUITY UPLC H-Class system (Waters) equipped with a CHIRALCEL OZ-RH chiral column (ϕ4.6 × 150 mm, 5 μm; Daicel Chemical Industry), a multiwavelength detector (ACQUITY UPLC PDA eλ detector; Waters), and a fluorescence detector (ACQUITY FLR detector; Waters). The HPLC conditions were as follows: mobile phase, 30% (*v*/*v*) acetonitrile in a 100 mM solution of potassium hexafluorophosphate in H_2_O; flow rate, 0.8 mL min^−1^; fluorescence detection, excitation/emission, 430/570 nm.

## 5. Conclusions

In this study, we successfully obtained (*R*)-CypL by chiral HPLC separation using a chiral column and for the first time demonstrated that the reaction of the chiral-separated (*R*)-CypL with a recombinant CypLase produced light. In addition, we found that the maximum luminescence intensity from the reaction of (*R*)-CypL with CypLase was significantly lower than that from the reaction of (*S*)-CypL with CypLase, but our kinetic analysis of CypLase suggested a higher affinity of (*R*)-CypL for CypLase than (*S*)-CypL. Furthermore, we showed that CypLase consumed (*R*)-CypL more slowly than (*S*)-CypL. This result indicates that the slower turnover rate of CypLase for (*R*)-CypL was probably the cause of the luminescence of (*R*)-CypL with CypLase being less efficient than that of (*S*)-CypL with CypLase. These findings provide a new insight into the engineering of CypLase to produce light efficiently. A future study such as crystallographic characterization of CypLase with CypL, CypOxyL, or CypL analogs would be expected to reveal the enzymological reason for the differences in luminescence between (*R*)- and (*S*)-CypL with CypLase.

## Data Availability

The data presented in this study are available from the corresponding author on reasonable request.

## Supplementary Materials

The supporting information can be downloaded at: https://www.mdpi.com/article/10.3390/ijms25052699/s1. Reference [54] is cited in the supplementary materials.

## Abbreviations

AGP	Alpha 1-acid glycoprotein
CCD	Charge-coupled device
CypLase	*Cypridina* luciferase
CypL	Cypridina luciferin
HCl	Hydrochloric acid
HPLC	High-performance liquid chromatography
NMWL	Nominal molecular weight limit
CypOxyL	Cypridina oxyluciferin
rlu	Relative light unit
RT	Retention time
Tris	Tris(hydroxymethyl)aminomethane

## Appendix A

**Table A1:** The ratio of the partially integrated luminescence intensity of (*R*)- and (*S*)-CypL with CypLase to integration over 120 min in the luminescence measurements shown in Figure 4b,c.

Concentration of the Substrate	Substrate	The Ratio of the Partially Integrated Luminescence Intensity to the Integration over 120 min	
		0–12 min	12–120 min
0.1 μM	(*R*)-CypL	43.0%	57.0%
	(*S*)-CypL	51.2%	48.8%
1 μM	(*R*)-CypL	35.6%	64.4%
	(*S*)-CypL	49.5%	50.5%

**Table A2:** K_m_ values of CypLase for (*R*)- and (*S*)-CypL.

Substrate	K_m_ Value of CypLase
(*R*)-CypL	0.23 μM
(*S*)-CypL	0.75 μM

**Table A3:** The ratio of the partially integrated luminescence intensity of (*R*)- and (*S*)-CypL with human AGP to integration over 180 min in the luminescence measurements shown in Figure A2b,c.

Concentration of the Substrate	Substrate	The Ratio of the Partially Integrated Luminescence Intensity to the Integration over 180 min	
		0–18 min	18–180 min
0.1 μM	(*R*)-CypL	28.7%	71.3%
	(*S*)-CypL	29.5%	70.5%
1 μM	(*R*)-CypL	26.6%	73.4%
	(*S*)-CypL	26.6%	73.4%

**Table A4:** The ratio of the peak area of (*R*)- and (*S*)-CypL to the total CypL in the chiral HPLC analysis of the reaction mixtures shown in Figure 5.

Reaction Time	CypLase	The Ratio of the Peak Area of (*R*)- and (*S*)-CypL to the Total CypL	
		(*R*)-CypL	(*S*)-CypL
2 min	+	80.9%	19.1%
	-	56.7%	43.3%
7 min	+	94.6%	5.4%
	-	56.7%	43.3%
17 min	+	N.D. ^1^	N.D. ^1^
	-	56.7%	43.3%

^1^ N.D. indicates “not detected”.
