# Supplementary material for: Bioluminescence of (R)-Cypridina Luciferin with Cypridina Luciferase
Source: Int J Mol Sci. 2024 Feb 26;25(5):2699. doi: 10.3390/ijms25052699 (PMC10932282; doi:10.3390/ijms25052699)
Supplement: Supplementary file 1 [file ijms-25-02699-s001.zip › ijms-2836749-supplementary.pdf]

Supplementary information

Bioluminescence of (*R*)-Cypridina Luciferin with *Cypridina* Luciferase

Shusei Kanie [1,*,†], Chun Wu [2,†], Kiyohito Kihira [3], Rie Yasuno [4], Yasuo Mitani [1,*] and Yoshihiro Ohmiya [2,5]

[1] Bioproduction Research Institute, National Institute of Advanced Industrial Science and Technology (AIST), Hokkaido Center, 2-17-2-1 Tsukisamu-Higashi, Toyohira-ku, Sapporo 062-8517, Japan
[2] Biomedical Research Institute, AIST, Kansai Center, 1-8-31 Midorigaoka, Ikeda 563-8577, Japan
[3] Japan Aerospace Exploration Agency (JAXA), Tsukuba Space Center, 2-1-1 Sengen, Tsukuba 305-8505, Japan
[4] Cellular and Molecular Biotechnology Research Institute, AIST, Tsukuba Center, 1-1-1 Higashi, Tsukuba 305-8566, Japan
[5] Department of Biomedical Engineering, Osaka Institute of Technology (OIT), 5-16-1 Ohmiya, Asahi-ku, Osaka 535-8585, Japan
* Correspondence: s-kanie@aist.go.jp (S.K.); mitani-y@aist.go.jp (Y.M.)
† These authors contributed equally to this work.

Supplementary methods

SDS-PAGE analysis of a recombinant CypLase

SDS-PAGE analysis was performed using a 5% to 20% gradient of polyacrylamide gels (CHR520L; ATTO) and an EzRun SDS-PAGE running buffer (AE-1410; ATTO), in accordance with the manufacturer's protocol. Electrophoresis was run at a current of 21 mA for 27 min using a cPAGE Ace Twin electrophoresis device (WSE-1025; ATTO), and the resultant gel was stained with Coomassie Brilliant Blue solution (EzStainAQua; ATTO).

NMR analysis of CypL

Proton nuclear magnetic resonance (^{1}H NMR) spectrum was recorded on an AVANCE NEO (400 MHz) NMR spectrometer (Bruker, Billerica, MA, USA). The NMR chemical shifts (ppm) were referenced to the tetramethylsilane (TMS) peak. Data were reported as follows: chemical shift, multiplicity (s = singlet, d = doublet, t = triplet), coupling constant, and integration.

Supplementary figures

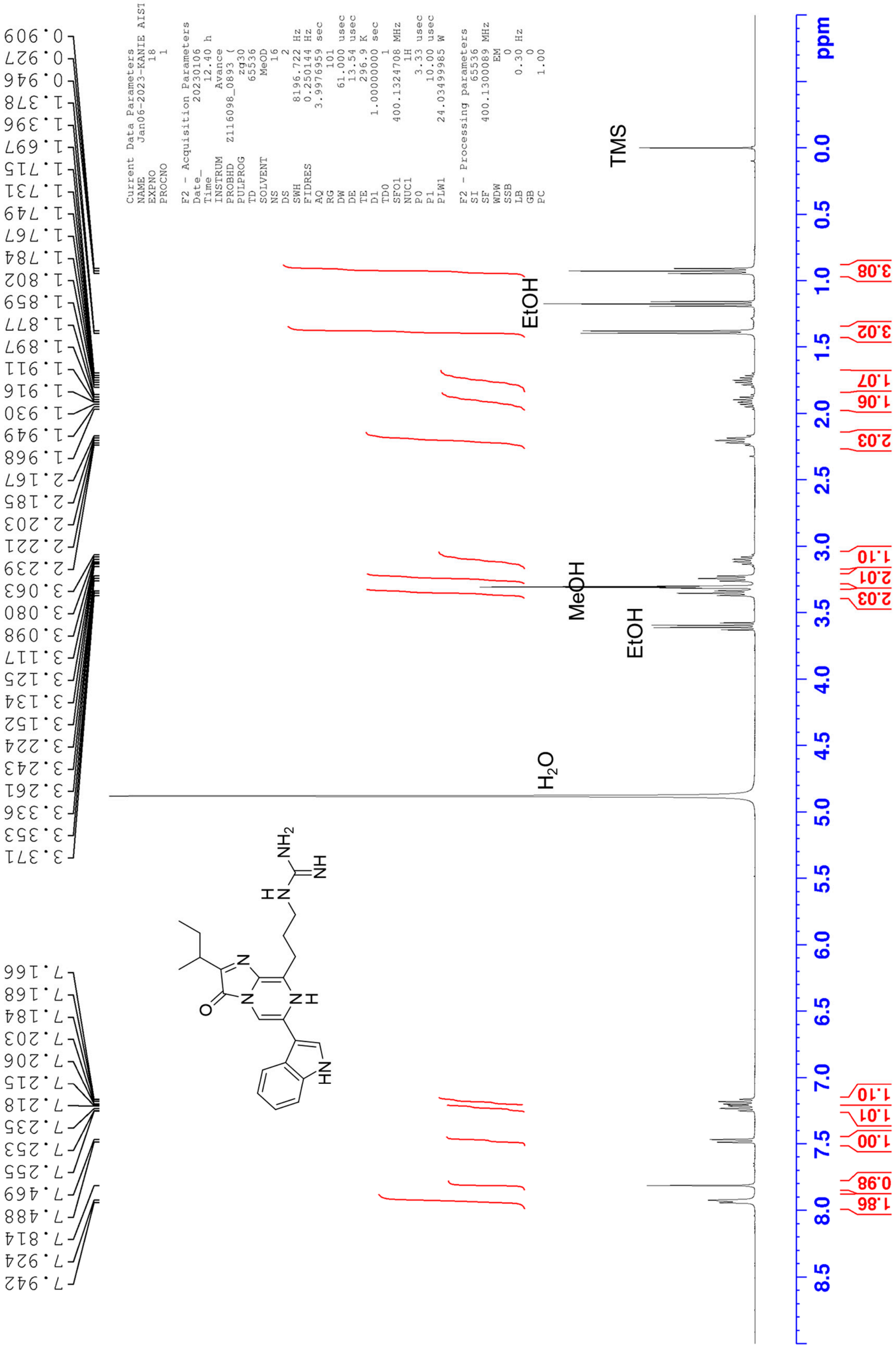

Supplementary Figure S1. ^{1}H NMR spectrum for CypL (NanoLight Technology, Prolume) used for chiral HPLC separation (400 MHz, CD_3OD).

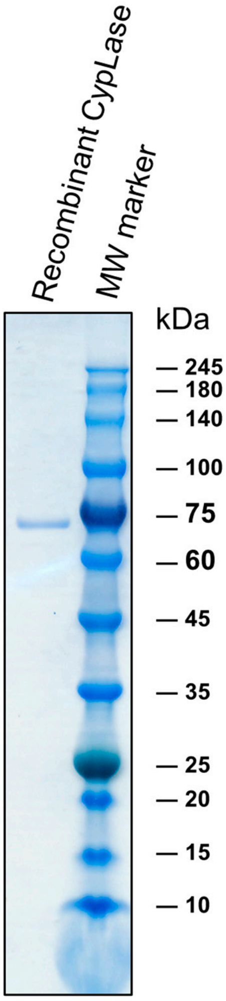

Supplementary Figure S2. SDS-PAGE analysis of a recombinant CypLase used in this study. The gel was stained with Coomassie Brilliant Blue solution (EzStainAQua; ATTO). The net volume of a 25 μg ml^{-1} solution of a recombinant CypLase loaded on the gel was 2.5 μL. MW marker, molecular weight marker (EzProtein Ladder; ATTO).

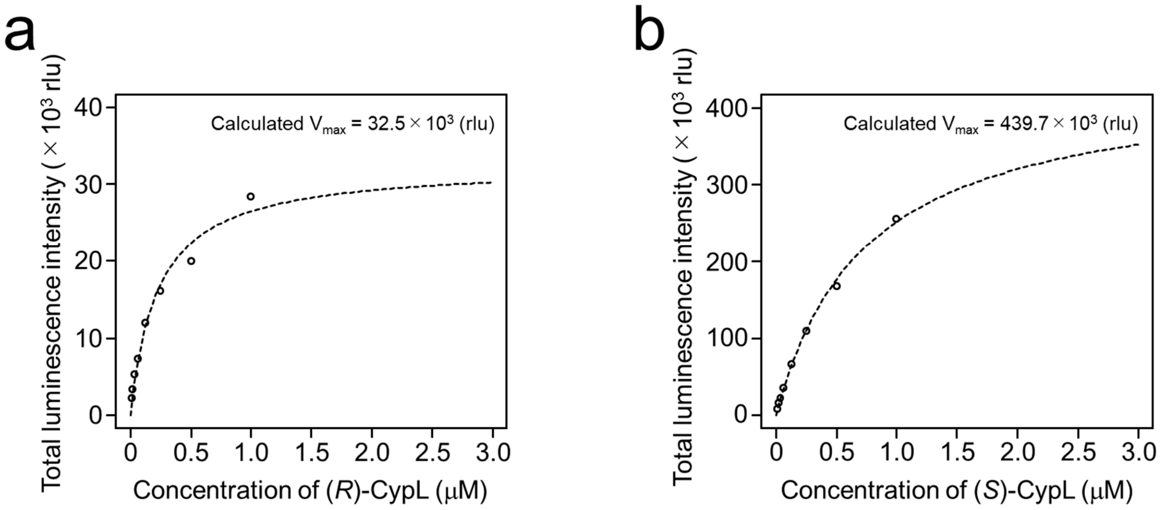

Supplementary Figure S3. Luminescence intensity of various concentrations of CypL with a recombinant CypLase. (a) (*R*)-CypL and (b) (*S*)-CypL with a recombinant CypLase (see Section 4 "Kinetic analysis of CypLase" in main text). The plotted values are average values.

Supplementary Table

Supplementary Table S1. ^{1}H NMR data for CypL (NanoLight Technology, Prolume) used for chiral HPLC separation (400 MHz, CD_3OD).

**data in the literature [54] (500MHz, CD_3OD) δ_H (integration, multi, *J* in Hz)**	**data in this study δ_H (integration, multi, *J* in Hz)**
0.91 (3H, t, 7.2)	0.93 (3H, t, 7.4)
1.36 (3H, d, 7.2)	1.39 (3H, d, 7.0)
1.69-1.76 (1H, m)	1.70-1.80 (1H, m)
1.87-1.95 (1H, m)	1.86-1.97 (1H, m)
2.16 (2H, quintet, 7.2)	2.20 (2H, quintet, 7.2)
3.08 (1H, sextet, 7.2)	3.11 (1H, sextet, 7.1)
3.20 (2H, t, 7.2)	3.24 (2H, t, 7.5)
3.32 (2H, t, 7.2)	3.35 (2H, t, 7.0)
7.19 (1H, t, 7.2)	7.19 (1H, t, 7.5)
7.24 (1H, t, 7.2)	7.24 (1H, t, 7.4)
7.48 (1H, d, 7.2)	7.48 (1H, d, 7.8)
7.74 (1H, s)	7.81 (1H, s)
7.89 (1H, d, 7.2)	7.93 (1H, d, 7.3)
8.06 (1H, s)	7.93 (1H, s)
